# Deciphering factors driving soil microbial life‐history strategies in restored grasslands

**DOI:** 10.1002/imt2.66

**Published:** 2022-12-04

**Authors:** Yang Yang, Yanxing Dou, Baorong Wang, Zhijing Xue, Yunqiang Wang, Shaoshan An, Scott X. Chang

**Affiliations:** ^1^ State Key Laboratory of Loess and Quaternary Geology, Institute of Earth Environment, Chinese Academy of Sciences Xi'an China; ^2^ Chinese Academy of Sciences Center for Excellence in Quaternary Science and Global Change Xi'an China; ^3^ National Observation and Research Station of Earth Critical Zone on the Loess Plateau in Shaanxi Xi'an China; ^4^ State Key Laboratory of Soil Erosion and Dryland Farming on the Loess Plateau Northwest A&F University Yangling China; ^5^ College of Geography and Tourism Shaanxi Normal University Xi'an China; ^6^ Department of Renewable Resources University of Alberta Edmonton Canada

**Keywords:** chronosequence, high‐throughput sequencing, microbial communities, plant properties, resource availability

## Abstract

In macroecology, the concept of r‐ and K‐strategy has been widely applied, yet, there have been limited studies on microbial life‐history strategies in temperate grasslands using multiple sequencing approaches. Total phospholipid fatty acid (PLFA) analysis, high‐throughput meta‐genomic sequencing, and GeoChip technologies were used to examine the changes in microbial life‐history traits in a chronosequence of restored grasslands (1, 5, 10, 15, 25, and 30 years since restoration). Grassland restoration increased the relative abundances of Actinobacteria, Proteobacteria, and Bacteroidetes but reduced the relative abundances of Acidobacteria, Planctomycetes, and Chloroflexi. PLFA analysis revealed that grassland restoration reduced the fungi:bacteria and Gram‐positive:Gram‐negative bacteria ratios. Combined with the meta‐genomic data, we found that grassland restoration shifted microorganisms from oligotrophic (K‐) to copiotrophic (r‐) groups, consistent with the increased rRNA operon copy number of the microbial community. Structural equation modeling showed that soil properties positively (*p* < 0.05) while plant properties negatively (*p* < 0.05) affected microbial life‐history traits. We built a framework to highlight the importance of plant and soil properties in driving microbial life‐history traits during grassland restoration. Finally, by incorporating meta‐genomic and other microbiological data, this study showed that microbial life‐history traits support the idea that rRNA operon copy number is a trait that reflects resource availability to soil microorganisms.

## Highlights

- Grassland restoration increased and decreased abundances of r‐ and K‐strategists, respectively.
- Grassland restoration shifted soil microorganisms from oligotrophic to copiotrophic.
- Soil properties positively affected microbial life‐history traits.
- Plant properties negatively affected microbial life‐history traits.

## Graphical abstract

We concluded that the macroecological theory could be applied to soil microbial life‐history traits and also highlight the key effects of plant and soil properties on the changes in microbial strategies. These findings were suitable for this Journal (significantly advance the field of Microbial Ecology) and first proved that macroecological theory could be applied to soil microbial community, which was benefit to the development of microbial ecology and can be regarded as the evidence for most of researches.

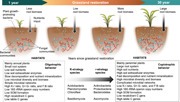

## INTRODUCTION

Microorganisms represent the vast majority of organisms that live in the soil and possess tremendous complexity in the terrestrial ecosystem [1, 2]. On the basis of existing ecological theory, microbial ecologists proposed trait‐based classifications of soil microorganisms [3]. The copiotroph–oligotroph classification was considered analogous to the r‐ and K‐selection theory for plants and animals [4, 5]. This classification was mostly based on microbial substrate preferences, trophic strategy, and growth rates and has since been widely applied in various environmental contexts [6, 7]. Previous study had applied Competitor–Stress tolerator–Ruderal (Co‐S‐R) life‐history strategies to microbial systems, particularly in the context of anthropogenic environmental change [8]. Because the Co‐S‐R approach allows the classification of microorganisms employing mixed life strategies (e.g., CoS, SR, etc.), it has been widely used in microbial ecology research [9]. Recent efforts proposed a revised life history that builds on the work of the Co‐S‐R classification framework [10, 11], and proposed three main microbial life‐history strategies: high growth yield (Y), resource acquisition (A), and stress tolerance (S), or Y‐A‐S, along two main axes of environmental variations: resource availability and abiotic stress [12]. For instance, Y‐strategists efficiently convert monomeric substrates, such as glucose, into microbial biomass and, later, into microbial residues. In contrast, A‐strategists prevail in conditions with low resource availability, where microorganisms are under pressure to favor resource capture at the expense of growth.

Soil microorganisms can adapt to both nutrient‐rich and ‐poor environments, complicating the relationship between microbial activity and the fate of carbon (C) [13]. In soil systems, the growth and functions of soil microorganisms are critically affected by the quantity and quality of substances entering the soil. Thus, microbial communities use a variety of life strategies to organize and structure their responses, which impact soil C dynamics [14]. Previous studies suggest that microbial community composition is related to its effectiveness or substrate utilization strategy [15, 16]. On the basis of their C mineralization potential and growth rates, microorganisms can be classified into two ecologically functional categories, that is, r‐ and K‐strategists [17]. The r‐selected species (copiotrophic or opportunistic species) have a fast growth rate, low substrate affinity, and a rapid response to available C and nutrient inputs, and typically flourish in environments enriched in labile C [1, 2]. In contrast, K‐selected species (oligotrophic or equilibrium species) are slow growing, have a high substrate affinity, and are more efficient with decomposing recalcitrant C [1, 2]. To explain the linkages among microbial growth, C cycling, and resource acquisition, copiotrophs (r‐strategists) and oligotrophs (K‐strategists) have been used to predict the microbial activity and their environmental interactions, as these processes largely control C cycling.

With the advancement of meta‐genomic sequencing techniques, microbial life‐history traits have been widely applied in various ecosystems [18, 19]. On the basis of meta‐genomic sequencing, the rRNA operon copy number has been suggested as a community‐level microbial trait to identify r‐ or K‐strategists [18, 20]. In general, microorganisms with more rRNA operon copy numbers are broadly considered to be r‐strategies, because the number of rRNA operon is often correlated with the maximum growth rate, the ability to change growth rates, and fewer types of higher‐affinity transporters [21, 22]. The rapid growth of copiotrophic groups requires a substantial increase in cellular ribosome content, which is achieved by an increase in rRNA operon copy number in their genomes [23]. In contrast, microorganisms with one or a few rRNA operon copy numbers are considered to be K‐strategies, and adapted to extract maximum resources out of a limited supply [24, 25]. The efficient growth of oligotrophs leads to the greater production of offsprings per unit resource consumed, and drives gene loss and reduces the rRNA operon copy number. In addition, fast‐growing copiotrophs are also predicted to enhance the usage of synonymous codons in their ribosomal genes because they underwent translational selection, leading to greater codon usage bias [26, 27]. Therefore, greater codon usage bias is typically found to be associated with a higher maximum growth rate, a pivotal trait representing the ability of copiotrophs in response to resource pulses.

Land degradation, mainly caused by human activities, is widely distributed and is a major challenge for global environmental protection [28, 29]. The Loess Plateau of China is facing severe land degradation issues [30, 31], and is susceptible to erosion because of the highly heterogeneous terrains and man‐made interference [32, 33]. Since 1999, the Chinese government has launched the “Grain‐to‐Green” program to restore vegetation, control soil and water erosion, and preserve the ecological environment [30, 34]. Grassland restoration is widely used for revegetation in Northwest China [35, 36]. These initiatives alter the structure and composition of soil microbial communities [37, 38, 39, 40]. Due to different methods used to study microbial life‐history strategies, soil microorganisms presented different life‐history strategies during vegetation restoration. Previous meta‐analysis and experimental studies have shown significant increases in the fungi‐to‐bacteria ratio during the natural succession of abandoned cropland [39, 41, 42], because bacteria were often regarded as r‐strategists and fungi as K‐strategists. For example, Zechmeister‐Boltenstern [43] found that bacterial communities shifted from r‐ to K‐strategist groups during vegetation succession, with fungal abundances, and the ratio of fungi to bacteria increased with succession. Similarly, at a finer taxonomic scale, soil microbial communities tended to shift from r‐ to K‐strategists, both at the phylum and genus levels in a secondary succession of *Quercus liaotungensis* forests. [44]. While Zhang et al. [45] reported that bacterial communities shifted from Acidobacteria‐predominant (slow‐growing oligotrophic groups, K‐strategists) to Proteobacteria‐predominant communities (fast‐growing copiotrophic groups, r‐strategists) in a 30‐year vegetation succession. Thus, soil microbial life‐history strategies were widely different among different ecosystem types, as revealed by the sequencing approach. However, research on microbial life‐history strategies studied with multiple sequencing approaches is lacking in temperate grasslands. Improving our understanding of the influence of plant and soil properties on the life‐history traits of microorganisms is essential for unraveling how they regulate the biogeochemical cycles in terrestrial ecosystems.

**Figure 1:**
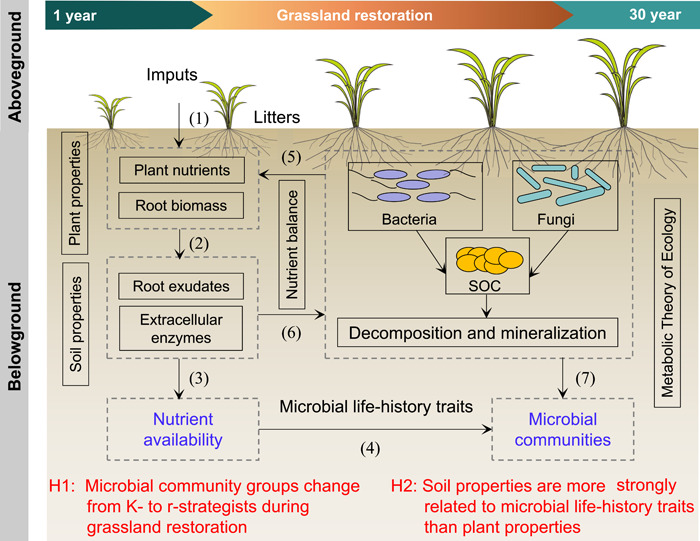
A conceptual framework showing the impact of plant and soil properties on microbial life‐history traits by nutrient acquisition during grassland restoration. Soil organic carbon (SOC) is mainly formed by microbial transformations of plant litter, and soil bacteria and fungi are large contributors to the decomposition and mineralization of SOC. In the process of grassland restoration, plant litter is decomposed by a wide range of bacteria and fungi (1) and various root exudates and extracellular enzymes are produced (2) making nutrients available for uptake by microorganisms (3), and then shaped microbial community composition (4). The process of nutrient element release and absorption. Most of the microorganisms have access to organic nutrients and deliver these nutrients to plants, which keep the nutrient balance (5). In turn, the available nutrients promote the growth and reproduction of microorganisms (6, 7), thereby making them available to plants, enhancing plant growth during grassland restoration.

A conceptual model was established to show the coeffects of plant and soil properties on microbial life‐history strategies during grassland restoration (Figure 1). Soil organic carbon (SOC) is formed by microbial transformation from plant litter, and soil bacteria and fungi are the main contributing factors to the decomposition of SOC, depending on nutrient availability [18, 20]. Grassland restoration changes the local environmental conditions through direct modification of litter, root systems, root exudates, and soil properties [46, 47]. As grassland restoration proceeds, plant roots produce several signaling substances and chemical compounds to protect themselves [48, 49, 50]. Most of the compounds in root exudates can promote the production of microbial biomass and extracellular enzymes [43, 47], and the decomposition of SOC is enhanced by these extracellular enzymes [51, 52, 53]. This leads to a shift in the composition and structure of soil microorganisms, ultimately altering the microbial life‐history traits [54, 55]. Here, community‐level traits inferred from meta‐genomic data can be used to distinguish copiotrophic and oligotrophic microbial communities. Especially, the r‐ and K‐strategy classification scheme might need to move beyond the restrictive focus on taxonomic genes [50], and thus we suggested expanding this theory to include data sets of functional genes. That is, functional genes involved in the degradation of labile and recalcitrant C could be grouped into r‐ and K‐strategy categories, respectively [50]. This way, these life‐history traits allow us to directly associate microbial performance with the related functional genes during grassland restoration. Thus, we used soil 16S rRNA, and internal transcribed spacer (ITS) rRNA gene sequencing to quantify the (relative) abundance, while using meta‐genomic sequencing to analyze the functional gene data to explain the special microbial life‐history traits during long‐term grassland restoration (Supporting Information Figure S1). The following two hypotheses were tested: H1, soil microbial community groups would change from K‐ to r‐strategists as the time since grassland restoration increases; and H2, soil properties are more strongly related to microbial life‐history traits than plant properties.

## RESULTS

### Vegetation community composition and soil properties

At the early restoration stage (mainly the 1‐, 5‐, and 10‐year sites), annual herbaceous plants (e.g., *Artemisia capillaris* and *Artemisia scoparia*) were the dominant species with low soil nutrients, microbial biomass, and enzyme activities (Table 1). At the late restoration stage (>10 years), perennial plants (e.g., *Stipa bungeana* and *Artemisia stechmanniana*) were the dominant species (Supporting Information Table S1), with high root biomass, soil nutrients, and enzyme activities (Table 1). The leaf C, N contents and root biomass gradually increased with years since grassland restoration. Soil properties, including soil organic C (SOC), moisture content (SM), total N (STN), available P (SAP), microbial biomass C (MBC), N (MBN), acid phosphatase (AP), *α*‐1,4‐glucosidase (AG), and *β*‐*N*‐acetylglucosaminidase (NAG), gradually increased with the year since grassland restoration and peaked at the 30‐year site, but the opposite trend was found for soil pH and microbial biomass P (MBP).

**Table 1:** Effects of grassland restoration on plant and soil properties (means ± standard error)

	Year since restoration (yr)					
Item	1	5	10	15	25	30
*Plant properties*						
Root biomass (g m^−2^)	12.4 ± 1.5 d	19.3 ± 2.3 c	23.5 ± 2.4 c	37.9 ± 2.1 b	41.7 ± 3.5 b	57.8 ± 5.3 a
Root C (g kg^−1^)	288 ± 24 e	324 ± 35 d	336 ± 26 d	357 ± 17 c	384 ± 28 b	398 ± 23 a
Root N (g kg^−1^)	0.76 ± 0.05 a	0.74 ± 0.07 a	0.74 ± 0.04 a	0.75 ± 0.06 a	0.73 ± 0.03 a	0.62 ± 0.05 b
Root P (g kg^−1^)	0.38 ± 0.02 a	0.44 ± 0.03 c	0.45 ± 0.05 c	0.48 ± 0.04 b	0.53 ± 0.06 b	0.64 ± 0.05 a
Leaf C (g kg^−1^)	439 ± 23 d	446 ± 52 c	458 ± 43 c	461 ± 27 b	467 ± 35 b	472 ± 36 a
Leaf N (g kg^−1^)	1.58 ± 0.24 a	1.57 ± 0.26 a	1.54 ± 0.18 a	1.55 ± 0.33 a	1.48 ± 0.41 a	1.45 ± 0.52 a
Leaf P (g kg^−1^)	0.28 ± 0.03 a	0.28 ± 0.02 a	0.30 ± 0.02 a	0.31 ± 0.01 a	0.32 ± 0.03 a	0.33 ± 0.02 a
*Soil properties*						
pH	7.73 ± 0.23 a	7.21 ± 0.24 b	7.02 ± 0.18 b	6.54 ± 0.37 c	6.17 ± 0.21 c	6.03 ± 0.25 d
SM (%)	9.42 ± 2.14 d	10.31 ± 2.75 c	11.62 ± 1.95 c	12.93 ± 3.16 b	13.11 ± 3.15 b	14.20 ± 2.22 a
SOC (g kg^−1^)	9.12 ± 1.89	10.36 ± 2.13	12.52 ± 2.56	13.51 ± 2.34	15.68 ± 1.89	16.17 ± 2.45
STN (g kg^−1^)	1.22 ± 0.23 d	1.32 ± 0.35 c	1.35 ± 0.26 c	1.48 ± 0.17 b	1.53 ± 0.35 b	1.59 ± 0.21 a
SAP (mg kg^−1^)	23.4 ± 3.4 e	25.8 ± 2.8 e	36.8 ± 5.4 d	43.2 ± 4.1 c	62.6 ± 5.4 b	68.7 ± 5.9 a
MBC (mg kg^−1^)	341 ± 36 d	355 ± 39 c	362 ± 26 c	389 ± 42 b	403 ± 49 b	422 ± 37 a
MBN (mg kg^−1^)	87.1 ± 8.6 d	92.1 ± 9.6 c	98.0 ± 11.2 c	101.1 ± 7.9 b	109.0 ± 9.2 b	115.2 ± 15.1 a
MBP (mg kg^−1^)	19.2 ± 3.1 a	18.8 ± 5.2 a	18.2 ± 2.1 a	17.9 ± 3.1 a	17.6 ± 2.5 a	17.3 ± 3.8 a

*Note*: Different lowercase letters indicate a significant difference (*p* < 0.05) among the treatments (year since restoration) by Fisher's test following the analysis of variance.

Abbreviations: MBC, microbial biomass C; MBN, microbial biomass N; MBP, microbial biomass P; SAP, soil available P; SM, soil moisture content; SOC, soil organic C; STN, soil total N.

### Soil microbial community structure

Soil bacterial sequences were classified into 13 phyla at the 97% level (Supporting Information Table S2), and the dominant phyla (relative abundance >5%) were Actinobacteria, Acidobacteria, and Proteobacteria. Soil fungal sequences were classified into eight phyla at the 97% level (Supporting Information Table S3), and the dominant phyla (relative abundance >5%) were Ascomycota, Basidiomycota, and Chytridiomycota. The relative abundances of Actinobacteria, Proteobacteria, and Bacteroidetes were higher at the 30‐year site and subsequently increased with the year since grassland restoration, while Acidobacteria, Planctomycetes, and Chloroflexi had high relative abundance at the 1‐year site, and then decreased with the year since grassland restoration (Supporting Information Table S4). For soil fungal phyla, Basidiomycota had a high relative abundance at the 1‐year site, and the abundance increased with the year since grassland restoration, while Ascomycota had a high relative abundance at the 30‐year site, and the abundance decreased with the year since grassland restoration (Supporting Information Table S5).

**Figure 2:**
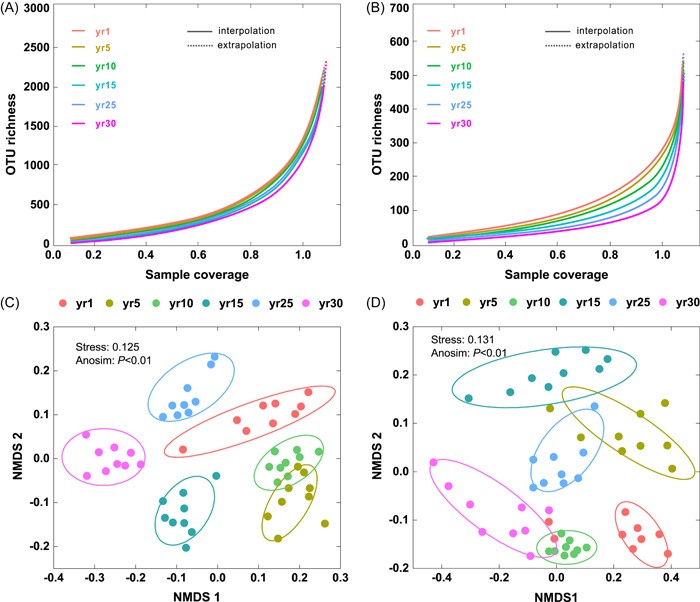
Coverage‐based rarefaction (solid line segment) and extrapolation (dotted line segments) sampling curves of bacteria (A) and fungi (B) during grassland restoration. Nonmetric multidimensional scaling (NMDS) representation of (C) soil bacterial communities and (D) fungal communities with year since grassland restoration. Operational taxonomic units (OTUs) at 97% similarity defined the communities, and ordinations were based on Bray–Curtis dissimilarities.

The bacterial (Supporting Information Table S2) and fungal (Supporting Information Table S3) Shannon–Wiener indices were the lowest at the 1‐year site and increased with the age of the restored grassland, reaching a maximum at the 30‐year site. We obtained smooth and saturated rarefaction curves as the sequence number increased with 97% similarity (Figure 2A,B), and bacterial and fungal communities showed a different classification according to nonmetric multidimensional scaling (NMDS; Figure 2C,D). The fungal communities displayed greater phylogenetic distances than bacterial communities (Supporting Information Figure S2).

Network analysis (Supporting Information Figure S3) shows that there were more positive than negative correlations in all networks during grassland restoration. The networks of the soil microbial community at the 30‐year site were stronger (more abundant nodes) than at other sites. We found a typical module structure due to the calculated modularity index, which was more than 0.4 (Supporting Information Table S6). The average clustering coefficient (avgCC), and the values of the average path length in these empirical networks gradually increased with year since restoration. In addition, the modularity index and average connectivity (avgK) gradually increased with year since restoration, while the average path length and network diameter showed the opposite trend.

### Soil microbial life‐history traits and their functional genes

**Figure 3:**
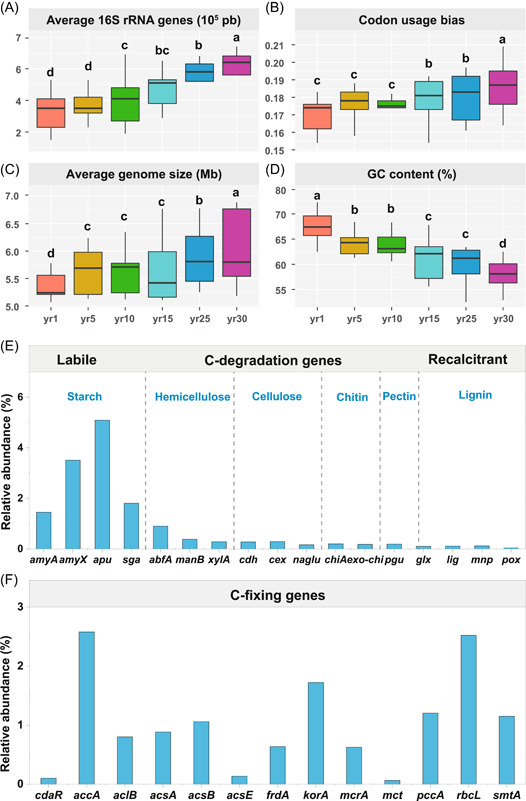
Soil microbial life‐history traits during grassland restoration. Box plots show the comparisons of life‐history trait values during grassland restoration. The medians in these box plots are as follows: average 16S rRNA genes (A), codon usage bias (B), average genome size (C), and GC content (D). The lower and upper boundaries of the box represent the first and third quartiles, respectively; the horizontal line represents the mean; the bounds of the lower and upper bars reflect the 10th and 90th percentiles, respectively. Different lowercase letters indicate a significant difference (*p* < 0.05) among the treatments (year since restoration) by Fisher's test following the analysis of variance. Relative abundance of soil microbial functional genes (E, C degradation genes; F, C fixation genes) during grassland restoration. The genes are arranged by the biodegradability of their target substrates, from labile to recalcitrant. GC, guanine–cytosine.

The average 16S rRNA genes, codon usage bias, average genome size gradually increased with the year since restoration (Figure 3A–D), whereas variation in guanine–cytosine (GC) contents was found to follow a reverse pattern. No significant difference in 16S rRNA genes and codon usage bias was found between 1‐ and 5‐year sites (*p* > 0.05).

We analyzed 17 gene families encoding enzymes for C degradation and fixation (Figure 3E,F). Among C degrading genes, the relative abundances in *amyA* (encoding *α*‐amylase), *amyX* (encoding glucoamylase), *abfA* (encoding hemicellulose), and *apu* (encoding amylopectin) increased, while *glx* (encoding glyoxal oxidase for lignin degradation) decreased with year since restoration (Supporting Information Table S7). The relative abundances of the genes for C fixation (*accA*, *aclB*, *acsA*, and *rbcL*) and those of *acsB* and *smtA* gradually decreased with year since restoration (Supporting Information Table S8).

**Figure 4:**
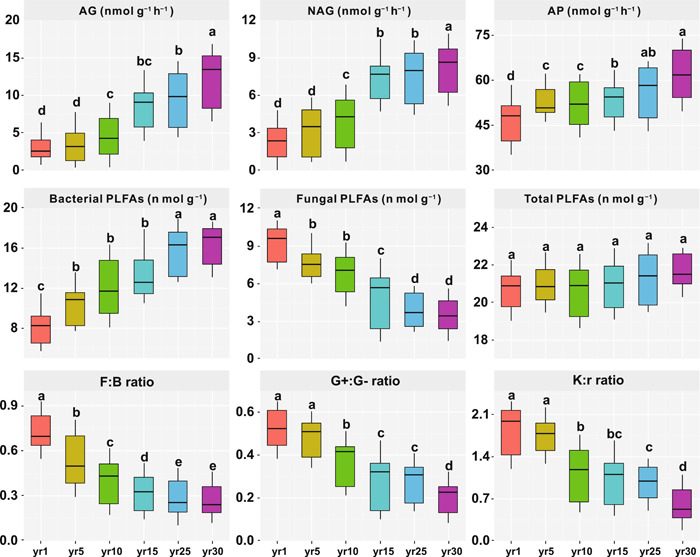
The response of major soil extracellular enzyme activities and microbial groups (indicated by PLFAs) during grassland restoration. All values are presented as means (standard errors). Different lowercase letters indicate a significant difference (*p* < 0.05) among the treatments (year since restoration) by Fisher's test following the analysis of variance. AG, *α*‐1,4‐glucosidase; AP, acid phosphatase; F:B ratio, fungal‐to‐bacterial PLFA ratio; G+:G− ratio, Gram‐positive to Gram‐negative bacterial PLFA ratio; NAG, *β*‐*N*‐acetylglucosaminidase; PLFAs, phospholipid fatty acids.

Fungal phospholipid fatty acids (PLFAs) gradually reduced, while bacterial PLFAs gradually increased with year since restoration (Figure 4); however, grassland restoration had no significant effect on the total PLFAs (*p* > 0.05). Meanwhile, fungal‐to‐bacterial PLFA ratio, Gram‐positive to Gram‐negative bacterial PLFA ratio, and K:r ratio gradually decreased with year since restoration.

### Importance of plant and soil properties on microbial life‐history traits

**Figure 5:**
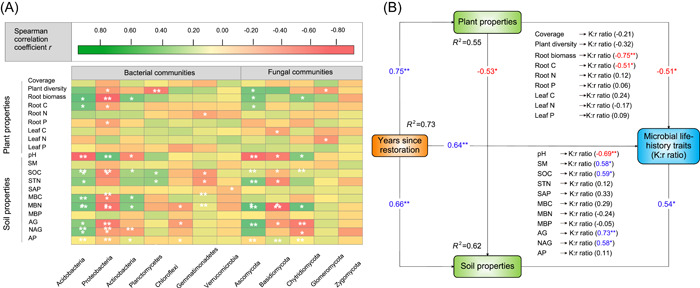
Spearman correlation analysis between the relative abundance of dominant soil microbial phyla (left columns, bacterial communities; right columns, fungal communities) and plant and soil properties (A). The color of each cell is proportional to the value of Spearman's correlation coefficient. Green indicates a positive correlation (dark green, *r* = 0.80); red indicates a negative correlation (dark red, *r* = 0.80). *p < 0.05; **p < 0.01. The final structural equation model (SEM) depicting the effects of plant and soil properties on microbial life‐history traits (*p* = 0.634; *χ* ^2^ = 6.12; GFI = 0.984; AIC = 156.23; RSMEA = 0.002, 75%) (B). The rectangles are including observable variables. The numbers adjacent to the arrows are standardized path coefficients, analogous to relative regression weights, and are indicative of the effect of the size on the relationship. *R* ^2^ denotes the proportion of variance explained. The red numbers mean the negative effect, and the blue numbers mean the positive effect, respectively. AG, *α*‐1,4‐glucosidase; AP, acid phosphatase; MBC, microbial biomass C; MBN, microbial biomass N; MBP, microbial biomass P; NAG, *β*‐*N*‐acetylglucosaminidase; SAP, soil available P; SM, soil moisture content; SOC, soil organic C; STN, soil total N.

The relative abundance of Acidobacteria was positively correlated with root biomass, root C, STN, MBC, MBN, AG, and NAG (*p* < 0.05; Figure 5A). In contrast, the relative abundance of Proteobacteria was related to soil pH and negatively correlated with root biomass, root C, SOC, STN, MBC, MBN, and AG. For soil fungal phyla, the relative abundance of Ascomycota was positively correlated with root biomass, root C, STN, MBN, AG, and NAG. The relative abundance of Basidiomycota was negatively related to soil pH.

According to the final model in our structural equation modeling, soil properties positively while plant properties negatively influenced microbial life‐history traits (*p* < 0.05; Figure 5B). Most importantly, the variance of the changes in microbial life‐history traits was explained better by soil properties than by plant properties (Figure 5B).

## DISCUSSION

### Effects of grassland restoration on soil microbial communities

Generally, soil bacteria are classified as r‐strategists, and soil fungi are described as K‐strategists [35, 40, 45, 50, 56, 57, 58, 59]. Previous researches have shown a significant increase in soil fungi‐to‐bacteria ratio during the natural succession of abandoned croplands, indicating a possible shift from r‐ to K‐strategists [56, 57, 58, 59]. However, in this study, the ratio of fungal‐to‐bacterial biomass decreased during grassland restoration (Figure 4), which is consistent with other work on vegetation succession, in which bacteria may outcompete fungi during vegetation succession [60, 61, 62]. This is because soil fungi, especially filamentous fungal species, have a greater capacity than bacteria to explore the soil's pore networks at macroscales [63, 64]. Consumption of labile C compounds by bacterial r‐strategists at the early restoration stage might therefore be limited by short‐distance transport [54, 55, 65]. This limitation was lifted with grassland restoration, leading to the increase in bacterial r‐strategists. In addition, the ratio of Gram‐positive (G+):Gram‐negative (G‐) bacteria ratio also decreased during grassland restoration (Figure 4). Gram‐negative bacteria prefer to utilize more labile organic C than Gram‐positive bacteria. This leads to the decrease of K‐strategists, since the K‐strategists are more adapted to nutrient‐poor niches and efficient to mineralize the recalcitrant C [66, 67, 68, 69], implying a shift from K‐ towards r‐strategy and supporting our first hypothesis.

At the deep branching clades, Actinobacteria, Proteobacteria, Bacteroidetes, and Ascomycota are often considered fast‐growing copiotrophic members, which are endowed with numerous enzymes for the depolymerization of fresh organic matter and feed on labile organic C, and can be characterized as typical r‐strategists [70, 71, 72]. In this study, Actinobacteria, Proteobacteria, Bacteroidetes, and Ascomycota were particularly enriched in 30 years of restoration (Supporting Information Table S4), and all are rich in labile organic C. These copiotrophic microorganisms are typical soil inhabitants and respond fast to nutrient‐rich conditions, but are also able to utilize proteins and lipids (microbial components) and to participate in degrading polymers, such as cellulose [14, 60, 61, 62, 65, 73]. However, Acidobacteria, Planctomycetes, Chloroflexi, and Basidiomycota are relative K‐strategists (oligotrophs) as they can grow on hemicellulose or cellulose and mineralize recalcitrant organic C [60, 62, 73]. These slow‐growing or oligotrophic microorganisms play an important role in SOC decomposition because they primarily invest in the production of extracellular enzymes for the degradation of complex C during periods of resource scarcity [4, 5]. At an early restoration stage, low nutrient availability usually limits microbial growth and activity, in this case, microorganisms are dominant with r‐strategists, because they help in population re‐establishment after disturbance [51, 52]. Due to the poor availability of soil nutrients, these microorganisms usually invest more energy in reproduction than in growth, metabolism, or improving competitiveness [5, 74, 54]. However, this limitation can be alleviated as restoration continues over time because of increased soil C input and nutrient accumulation after vegetation restoration [1, 40]. At a late restoration stage, the relative abundance of Basidiomycota under the category of oligotrophs (K‐strategists) decreased. The rich soil nutrient resources were beneficial to microbial growth, and improved the decomposition and mineralization of organic matter, leading to high competition and stable populations (r‐strategists) [5, 19]. Such microorganisms with rapid growth stimulate the uptake of nutrients through root systems, which can generate more litter and root exudates, leading to a balance between nutrient cycling and decomposition [14, 54, 75, 76, 77].

In microbial networks (Supporting Information Figure S3), the high positive interactions of microbial co‐occurrence networks at the 30‐year site indicated that the proportion of potential cooperative interactions between microbial groups was relatively high [78, 79]. The high modularity of co‐occurrence network means that populations within communities may possess semblable ecological niches or modular structures [80, 81]. Compared with the 1‐year site, the microbial community network at the 30‐year site was more modular and had a more complex community structure (Supporting Information Figure S3). It is generally accepted that more complex communities have greater stability [1]. Therefore, the more complex microbial community network at the 30‐year site had higher stability when coping with a changing environment. This is because the higher soil nutrient resource increases the metabolic reaction of specific microorganisms, allowing them to occupy more ecological niches after long‐term environmental filtering [76]. In addition, fungi showed a higher Bray–Curtis distance than bacteria (Figure 2), indicating that soil fungi are more likely to mutate and evolve than bacterial during grassland restoration. This finding is consistent with other work on secondary succession [58], in which fungi may outcompete bacteria during succession and generate greater variation and evolution [82], because they establish close associations with plants and maybe more effective in utilizing the C available from plants.

### Effects of grassland restoration on soil microbial functional genes and life‐history traits

Due to the enormous phylogenetic and physiological diversity within each phylum, it is unlikely that an entire phylum would share common ecological roles [1]. For example, in the phylum Proteobacteria, β‐Proteobacteria exhibit copiotrophic attributes (r‐strategists); while α‐Proteobacteria may not be classified as r‐strategists. Similarly, some members of the phylum Actinobacteria (e.g., Actinomycetales) are likely able to depolymerize complex and recalcitrant C compounds (e.g., lignin, cellulose), and, thus, showed a tendency as a K‐strategist [4, 5]. For example, in studies conducted in natural grassland and forest ecosystems, Actinobacteria grow slowly and can survive in nutrient‐poor conditions, and thus are usually classified as K‐strategists [17]. By contrast, in nutrient addition experiments, Actinobacteria is widely found to be positively related to increased N availability and classified as r‐strategists [83]. In addition to counting the proportion of likely oligotrophs or copiotrophs, the weighted averaged rRNA operon copy number is a better indicator for the ecological strategy of a microbial community [1]. Given that the rRNA operon copy number can be conveniently estimated by environmental genomic sequences in DNA databases because the rRNA operon copy number is conserved with phylogeny [4, 5]. It has been shown that genomic signatures, such as codon usage bias indices and the rRNA operon copy number, could be used as proxies for microbial life‐history strategies, because copiotrophs (r‐strategists) often have a higher rRNA gene copy number, while oligotrophs (K‐strategists) often have a lower rRNA gene copy number [6, 84]. The average copy numbers of rRNA genes gradually increased with year since grassland restoration (Figure 4), and the rich resource availability increased the ribosomal content and promoted the growth of copiotrophic microbial groups, further supporting the dominance of r‐strategists during grassland restoration. Moreover, the microorganisms demonstrated a high codon usage bias for the ribosomal gene [85, 86], indicating a high expression of the genes for rapid growth during grassland restoration [4]. This is because soil microorganisms usually use more energy for reproduction than for growth, metabolism, or enhancing competitiveness (K‐strategists) at the early restoration stage, whereas microorganisms at the late restoration stage had a low reproductive rate and a high survival rate, leading to high competitiveness and a stable number of populations (r‐strategists) [3, 4, 18, 19, 74, 40, 45].

The r/K selection concept was expanded to the functional gene data in this study. A previous study found that vegetation succession increased the diversity of soil microbial communities and the abundance of soil microbial genes associated with C fixation, C degradation (*amyA*, *nplT*, *xylA*, *CDH*, and *glx*) [45]. Vegetation succession increased C input and promoted the gene abundance of recalcitrant C decomposition. Our results showed that the abundances of labile C genes (*amyA*, *amyX*, *apu*, and *sga*) were higher in the older sites (Supporting Information Table S7), while the recalcitrant C genes (*chiA*, *pgu*, and *glx*) were higher at the early stage of grassland restoration (Supporting Information Table S8). It means that the population of soil microorganisms had a low reproductive rate but a high survival rate, resulting in strong competitiveness and a stable population of K‐strategists at the late restoration stage [4, 5, 14], while soil microorganisms often spend more energy on reproduction than on growth, metabolism and improving competitiveness (r‐strategists) at the late restoration stage. It is well known that the functional genes in microorganisms can regulate the corresponding activity of soil enzymes, and extracellular enzymes are the direct driver of soil C cycling [40]. The higher abundance of C‐fixing genes we found is a benefit to soil C sequestration [58, 81]. In addition, soil microorganisms are commonly C‐limited, and the enhanced availability of C due to increased plant production in conjunction with higher soil C contents, may contribute to greater microbial biomass to form recalcitrant C and benefit SOC accumulation, consistent with our previous studies [40, 42].

### Effects of grassland restoration on the K:r ratio and the ecological implications on microbial life‐history traits

The negative relationship of K:r ratio with plant properties and the positive relationship with soil properties (*p* < 0.05, Figure 5B) support our second hypothesis. Our data thus suggest that changes in microbial life‐history traits were more strongly correlated with soil properties than with plant properties, similar to the findings of Zhang et al. [45] and Cui et al. [71]. This was because soil properties (including microbial biomass, nutrient contents, and extracellular enzyme activities) were improved during grassland restoration, and this considerably enhanced microbial metabolism and promoted microbial growth [40, 71, 87, 88, 89]. However, Mitchell et al. [90] found that the composition of plant communities was a better predictor for the microbial community than soil chemical properties in a boreal ecosystem. Similarly, Peay et al. [91] showed that the composition of fungal communities was more tightly associated with changes in plant communities in tropical forests. It is possible that those relationships are ecosystem‐specific. This was because the nutrient requirements (from plant and soil resources) for microorganisms varied greatly in different ecosystems [92, 93]. For example, in most grassland ecosystems, the nutrient requirements were mainly from plant residues, but microbial residues were the primary nutrient resource for microorganisms in most forest ecosystems [92].

**Figure 6:**
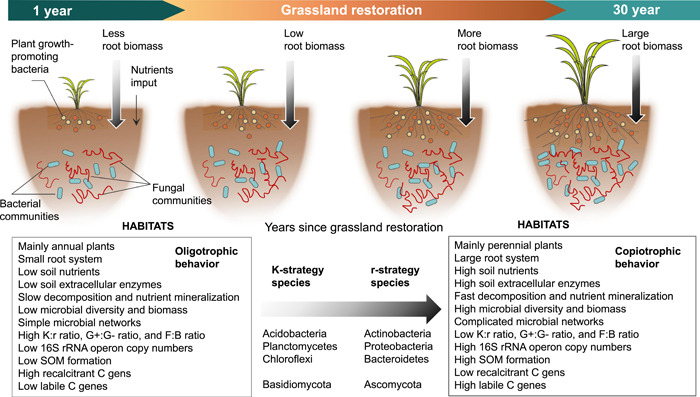
A proposed framework that reveals plant and soil properties driving the changes in microbial life‐history traits. According to the Monod equation and the resource availability (both concentration and loading rate), r‐strategists (copiotrophs or opportunistic species) are described as living in temporarily resource‐rich environments, and characterized by high nutritional requirements per unit cell mass, faster specific growth and weaker substrate affinity, which preferentially use easily available soil organic C (SOC) (e.g., root exudates and other low molecular weight or water‐soluble organic C sources) when resources are rich. By contrast, microbial K‐strategists (oligotrophs or equilibrium species) are described as having the opposite of these characteristics, with slower specific growth but stronger substrate affinity. During grassland restoration, the members with an r‐strategy gradually increase to improve community productivity and opportunity, while other members with a K‐strategy decrease due to adjustment due functional redundancy for enhanced community efficiency. A balance in community between r‐ and K‐strategies facilitates adaptation to resource disturbance during grassland restoration.

Here, we established a framework of how plant and soil properties drove changes in soil microbial life‐history traits during grassland restoration (Figure 6). This life‐history framework enables us to directly link microbial performance to nutrient resource availabilities. Our results show that grassland restoration can promote plant growth and then increase the root biomass (Table 1), which produces large amounts of exudates and organic resources for the growth of microorganisms in microhabitats [94, 95]. In this case, the improvement of the nutrient resource may also alter the relative abundances of microorganisms with different growth strategies [50, 66, 96, 97]. We demonstrated that multiple ecologically informed traits inferred from metagenomic data could advance the trait‐based characterization of microbial communities within a copiotroph–oligotroph framework (Figure 6). However, it is important to note that metagenomic approaches are biased towards bacteria because of their dominance in the soil DNA pool [43, 98, 99]. Other important mediators of soil biogeochemical processes, such as fungi, soil viruses, and antibiotics, are not as well incorporated. Therefore, future studies should increase the sample size, better integrate soil eukaryotes, viruses and antibiotics, and more precisely target specific biogeochemical processes are essential for a better understanding of the relationships between soil communities, genes, and processes.

## CONCLUSIONS

We conclude that multiple ecologically informed traits inferred from amplicon sequencing, meta‐genomic, and GeoChip data can be used to study microorganisms within a copiotroph–oligotroph framework, and soil microbial life‐history traits shifted with plant and soil properties. Soil microorganisms shifted from oligotrophic to copiotrophic groups during grassland restoration, which was more strongly related to soil than plant properties. Therefore, we further conclude that the macro‐ecological theory could be applied to soil microbial life‐history traits and highlight the key roles of plant properties (root biomass and C content) and soil properties (mainly soil pH, SOC, and extracellular enzymes) on the changes in microbial strategies in restored grasslands.

## METHODS

### Study site

This study was conducted in restored grasslands located at an altitude of 1800–2100 m on the Loess Plateau in China (Supporting Information Figure S1). The region has a temperate climate and is dominated by temperate grasslands. The annual precipitation is 400–450 mm, with 60–75% of the precipitation occurring from July to September. The annual evaporation is 1017–1739 mm, and the annual mean temperature is around 7.0°C, with approximately 2500 h of annual average sunlight.

Historically, grassland degradation was caused by overgrazing (>50 sheep ha^−1^), land cultivation, as well as excessive hay harvesting. Multiple measures (enclosure and no grazing) have been taken by the Chinese government to conserve grassland resources. Therefore, grazing was forbidden for the area for vegetation restoration after the 1980s. In the 1980s, a long‐term natural restoration experiment was commenced with the aim of restoring degraded grasslands in this area. Before grassland conservation, soil physicochemical properties in our study area with the same soil type and natural conditions were not significantly different. A range of grasslands with similar degradation levels was selected at random and fenced for natural restoration every few years, generating a 30‐year chronosequence of restored grasslands without livestock grazing and anthropogenic disturbance. Around 300 species of wild plants [40, 55], such as *A. stechmanniana*, *S. bungeana*, *Potentilla bifurca*, *Agropyron cristatum*, *Lespedeza daurica*, and *Thymus mongolicus*, were found in this region. Currently, this area is used as an experimental field to monitor grassland dynamics at the Chinese Academy of Sciences.

### Sampling design

On the basis of our previous studies in this area [40, 54], six grasslands representing 1, 5, 10, 15, 25, and 30 years since restoration were sampled in August 2019. These grasslands had similar elevation, slope position, and aspect, with the same typical loess soil [65]. Three random plots (40 × 60 m) were selected from each grassland. We installed three quadrats (1 × 1 m) in each plot (Supporting Information Figure S1). In each quadrat, five soil samples (more than 1 kg) were collected from the 0–10 cm depth using a stainless steel corer (5 cm inner diameter), and then mixed into one composite sample. Altogether, 6 × 3 × 3 = 54 samples were collected and sieved using a 2‐mm mesh to remove partial roots and debris.

A portion of soil samples was stored at 4°C for measuring microbial biomass carbon (C), nitrogen (N), phosphorus (P), and extracellular enzyme activities. After removing the litter horizon and biological crusts, soil samples for measuring microbial communities were collected in 50 ml centrifuge tubes. The samples were frozen in liquid nitrogen, placed in a box containing dry ice, and delivered to the laboratory. The tubes were stored at −80°C before DNA extraction. The samples that were used to detect soil physicochemical properties were sieved using a 2‐mm mesh and milled to powder (RM200, Retsch).

### Plant properties

In each quadrat, we cut all the plant shoots and dug up all the roots (1 × 1 m^2^) to a depth of 50 cm. We rinsed and dried the plant samples at 75°C till constant weight to determine the biomass. Then, the samples were sieved via a 0.5‐mm mesh to analyze C, N, and P concentrations. We also determined the vegetation composition in each quadrat (Supporting Information Table S1).

The root and leaf C, N concentrations were analyzed using an Element analyzer (AIC100). A microwave digestion system (NAI‐WB) was used to digest samples with 70% HNO_3_ for 12 h; leaf and root P concentrations were then measured by molybdenum blue colorimetry [100].

### Soil properties

We used a WET sensor (WET‐2 sensor, Delta‐T Devices Ltd.) for determining soil moisture content and a pH meter (Model PHS‐2, INESA Instrument) for measuring soil pH [101]. The Kjeldahl method was used for measuring STN, while the potassium dichromate external heating approach was used to measure SOC [102]. SAP was measured by treatment with 0.5 mol L^−1^ NaHCO_3_ followed by molybdenum blue colorimetry [101]. For measuring soil microbial biomass C, N, and P (MBC, MBN, and MBP, mg kg^−1^), we extracted approximately 4.0 g freshly prepared soil specimens with 0.5 M K_2_SO_4_ for a 1.5‐h period within the overhead shaker under ambient temperature, followed by filtration using paper filters (3 hw, Sartorius Stedim Biotech). Then, we determined MBC, MBN, and MBP using a fumigation–extraction method [101].

The MBC was calculated as EC/kEC, where EC = (organic C extracted from fumigated soils) − (organic C extracted from nonfumigated soils) and kEC = 0.45.
The MBN was calculated as EN/kEN, where EN = (total N extracted from fumigated soils) − (total N extracted from nonfumigated soils) and kEN = 0.54.
The MBP was calculated as EP/kEP, where EP = (total P extracted from fumigated soils) − (total P extracted from nonfumigated soils) and kEP = 0.40.

About 4.0 g of each fresh sample was stirred with 40 ml of 1 M acetic acid buffer solution (pH = 5.0) for 2 min, to obtain a homogenate. Next, 100 μl of this homogenate and 150 μl of acetic acid buffer solution were added to the wells of black 96‐well microplates. Compounds with high fluorescence, including 4‐methylumbelliferone (MUB), were used to measure extracellular soil enzymes, such as AP, AG, and NAG, by standard fluorometric approaches [18]. Then, the MUB standard curves (0, 2.5, 5, 10, 25, 50, and 100 μM) were plotted [48]. Each sample had a test (substrate + homogenate) and a control (buffer + homogenate), and each sample was assayed with eight replicates in the microplates. After being incubated at 25°C for 3 h, 1 μl of 1 M NaOH was added to stop the reactions in the samples. Fluorescence values were recorded at 450 nm for emission and 365 nm for excitation (Beckman Coulter DTX 880) [103]. After correcting for quenching, extracellular soil enzymes are expressed in units of nmol activity g^−1^ dry soil h^−1^ (nmol g^−1^ h^−1^). Enzyme commission numbers, incubation times, and substrates for each enzyme can be found in Supporting Information Table S9.

### Amplicon sequencing

Homogenized soil (0.5 g) was used for genomic DNA extraction with the Soil DNA Kit (200) (MoBio Laboratories). Primers were used for the V3–V4 regions of the 16S rRNA gene in soil bacteria exclusively by polymerase chain reaction (PCR) assay and included F515 (5′‐GTGCCAGCMGCCGCGGTAA‐3′) and R907 (5′‐CCGTCAATTCMTTTRAGTTT‐3′) for amplification [1]. For soil fungi, the ITS region, which was used as the universal DNA barcode marker, was amplified with the primers ITS1F (5′‐GGAAGTAAAAGTCGTAACAAGG‐3′) and ITS2R (5′‐GCTGCGTTCTTCATCGATGC‐3′). Following normalization with the purified amplicons, they were integrated at equimolar contents and underwent paired‐end sequencing (2 × 300 bp) on the Illumina MiSeq platform (Guhe Information Technology Co. Ltd.).

### Processing of sequence data

Using the Quantitative Insights Into Microbial Ecology (QIIME, version 2.0, https://qiime2.org/), we performed extra trimming and demultiplexing on sequence reads, and then the reads depth was corrected [78]. For the FastQ files, the Sickle software was used to filter raw sequence reads and remove sequences that were <150 bp or had a mean quality score <20. Then, after trimming the rest of the sequences to barcoded ITS or 16S rRNA gene sequences, we selected representative sequences by an online search using BLAST (http://sundarlab.ucdavis.edu/rice/blast/blast.html) according to the significant similarity. Then, QIIME was used to annotate the barcoded ITS or 16S rRNA gene sequences to various libraries and obtain operational taxonomic units (OTUs), which were screened by Vsearch (v. 1.11.1, https://github.com/torognes/vsearch/releases) for dereplication, clustering, and chimera detection. Taxonomy was assigned to OTUs based on their representative sequence by using Greengene 13.8 (http://www.greengene.com/). The number of sequences per sample was normalized by the size of the sequences and by the sampling coverage. Then, we performed coverage‐based rarefaction in R statistical software with the “iNEXT” package [79]. Indicators of soil microbial diversity, such as Shannon–Wiener, Simpson, and Chao1 indices, were measured by QIIME. The phylogenetic trees of the UniFrac distances among bacterial and fungal phylotypes were rooted and constructed using the neighbor‐joining method (Supporting Information Figure S2).

### Microbial PLFAs

Soil microbial PLFAs were extracted from each of 6 g freeze‐dried soil samples using a chloroform–methanol extraction mixture modified to incorporate a phosphate buffer [42]. The abundance of individual fatty acids was determined as nmol per gram dry soil. Standard nomenclature was used to describe PLFAs. PLFA concentrations were calculated based on the 19:0 (methyl nonadecanoate, C_20_H_40_O_2_) internal standard concentration. Overall, microbial community composition represented by PLFAs was distinguished into bacteria (Gram‐positive bacteria and Gram‐negative bacteria) and fungi, which are listed in Supporting Information Table S10. The G+:G− and F:B ratios represent Gram‐positive to Gram‐negative bacterial biomass ratio and fungal‐to‐bacterial biomass ratio, respectively.

In addition, the microbial life‐history strategies of K‐ and r‐members in microbial communities and functional genes are listed in Supporting Information Table S11. We used Gram‐positive bacteria and Gram‐negative bacteria to calculate the K:r ratio.

### GeoChip data and functional gene abundance

For determining the compositions of functional genes, the GeoChip 5.0 (60 K) technology was utilized to obtain the relative abundances of functional genes in soil microorganisms. There were ~60,000 oligonucleotide probes in the gene array belonging to ~400 gene families associated with biogeochemical events, such as C and N cycling genes [104]. In brief, after purification, this work labeled 0.8 μg DNA using Cy‐3 dye (GE Healthcare UK Limited), followed by purification using the QIAquick kit (Qiagen) as well as drying within the SpeedVac (ThermoSavant). After 24‐h hybridization of labeled samples under 67°C, this work utilized NimbleGen MS 200 microarray scanner (Roche) for scanning GeoChip microarray. Thereafter, Agilent's Data Extraction software was employed for image data processing and signal intensity transformation. We eliminated spots whose signal intensity was <2‐fold background, and the signal‐to‐noise ratio was <2 [105]. Additionally, probes measured within one replicate were eliminated. For every spot, we normalized its signal intensity based on relative abundances across diverse samples before later biostatistical analyses. The abundance of functional annotations was filtered, and annotations with a minimum abundance of 10% were removed. Finally, annotation abundance was normalized through log‐transformation [1, 3, 14].

### Metagenomic data and microbial life‐history traits

For meta‐genomic sequencing, the total soil genomic DNA was extracted using the FastDNA SPIN Kit (MP Biomedicals) and purified using the DNeasy PowerClean Pro Cleanup Kit (Qiagen) according to the guidelines of the manufacturers. The concentration of DNA in all the samples was obtained by equalization in PCR water (MoBio Laboratories), and then, the DNA was trimmed to 300 bp fragments by placing the samples in 50 µl tubes and subjecting them to an M220 Focused‐ultrasonicator (Covaris) for 90 s [25]. The DNA samples were subsequently transported to Weishengtai Technology Co. Ltd. for preparing the library and shotgun metagenomic sequencing using the Illumina HiSeq. 4000 platform (Illumina Inc.), following specific protocols (www.illumina.com). The reads were annotated using the subsystem and GenBank databases for functional genes and taxonomy, respectively [28]. The minimum identity of annotation filtering was 80%, with an e‐value cut‐off at 1 × 10^−5^ and a minimum alignment length of 20 bp, which was more restrictive compared to the default parameters [106].

We computed the sample traits based on metagenomic sequencing data to elucidate the life‐history traits of microorganisms. First, the rRNA operon copy number for each OTU was estimated through the rrnDB database, based on its closest relatives with known rRNA operon copy numbers. We calculated the abundance‐weighted average rRNA operon copy number of OTUs for each sample, taking the product of the estimated operon copy number and the relative abundance for each OTU and summing this value across all OTUs in a sample [107]. Second, Genome quantity was assumed to be the average coverage of single‐copy genes. We calculated the mean genome size as the quotient of the number of base pairs and genomes [108]. Third, we calculated the codon usage bias between the mean and upregulated genes [1]. Briefly, ΔENC' was used as an empirical estimator for selection strength acting on codon usage bias of upregulated genes. For every genome, the ΔENC' value was measured for all coding sequences (ΔENC'all) and ribosomal protein gene (ΔENC'rib) concentrations, with mean coding nucleotide frequency. Then, ΔENC' was indicated by

$$∆\mathrm{ENC}\prime =\frac{\mathrm{ENC}\prime_{\mathrm{all}}–\mathrm{ENC}\prime_{\mathrm{rib}}}{\mathrm{ENC}\prime_{\mathrm{all}}}.$$

For all gene sets, open reading frames of at least 450 bp were obtained using the EMBOSS function getorf [4]. For the upregulated gene sets, ribosomal proteins were obtained based on the similarity with the ribosomal proteins from a database for the existing sequenced genomes (e‐value < 10^–5^) [3, 109]. Finally, the genomic GC content was obtained from the NCBI Genome Database, and we calculated the variance of GC of the quality‐filtered reads as described in Wang et al. [47].

### Statistical analyses

Statistical analyses were conducted using the R software v. 3.4.2 (http://www.datavis.ca/R/), and one‐way analysis of variance combined with Fisher's test was used at 95% (*p* < 0.05) and 99% (*p* < 0.01) probability levels. We adopted NMDS based on Bray–Curtis dissimilarities to determine the changes in soil microbial community structures. The NMDS analysis was performed using the “vegan” package to visualize the sample relationships across different groups. Different network construction methods may lead to different information. SparCC was found to have the highest performance among a series of methods tested, which was also used to construct the co‐occurrence network in this study [79]. Co‐occurrence networks were constructed for microbial communities by Spearman correlations using the “corr.test” function in the psych R package. In each microbial taxa (bacteria or fungi) during grassland restoration, the 500 most abundant OTUs were selected to construct the SparCC co‐occurrence networks. Both Spearman correlation and SparCC results were filtered by the thresholds *r* > 0.75 and false discovery rate <0.05. Network graphs were generated by using the “igraph” R package, and the nodes in the co‐occurrence networks represent the OTUs, whereas the edges correspond to a significant correlation between nodes. To better quantify the topology of networks, a set of network parameters, including numbers of nodes and edges, average path length, network diameter, cumulative degree distribution, clustering coefficient, modularity, and so forth, was calculated and networks were visualized using the interactive platform Gephi 0.9.2 (https://gephi.org; Supporting Information Figure S3).

Spearman coefficients (using the “cor” function) were calculated to determine the correlation of environmental factors, such as plant and soil properties, and the relative abundance of the predominant microbial phyla, and then, heatmaps were made by using ORIGIN 21.0 software (http://www.origin.com/) [78]. The structural equation model (SEM) was particularly useful in large‐scale correlative studies, as it allows us to partition causal influences among multiple variables. The first step in SEM requires the establishment of an a priori model based on known effects on microbial life‐history traits (K:r ratio). A priori model (Supporting Information Figure S4) was established based on the effects of plant and soil properties on microbial life‐history traits (K:r ratio). This model included two groups: plant properties (including plant coverage, diversity, root biomass, root C, N, P, and leaf C, N, P) and soil properties (including soil pH, SOC, STN SAP, MBC, MBN, MBP, AG, NAG, and AP). Some data manipulation was required before modeling to improve the normality and linearity of our data. For example, data were transformed as necessary (including plant coverage, diversity, root biomass, root C, N, P, and leaf C, N, P, soil pH, SOC, STN SAP, MBC, MBN, MBP, AG, NAG, and AP) to meet assumptions of normality and homogeneity of variance, and tests were considered significant at *p* ≤ 0.05. Plant and soil properties were included as a composite variable. After attaining a satisfactory model fit, we introduced composite variables into our model. The use of composite variables does not alter the underlying model but collapses the effects of multiple conceptually related variables into a single‐composite effect, aiding the interpretation of model results [80]. Since some of the variables introduced were not normally distributed, the probability that a path coefficient differs from zero was tested using bootstrap resampling. Bootstrapping is preferred to the classical maximum‐likelihood estimation in these cases because, in bootstrapping, probability assessments are not based on the assumption that data follow a particular theoretical distribution [81]. Thus, data are randomly sampled with replacement to arrive at estimates of standard errors that are empirically associated with the distribution of the data found in the samples. When these data manipulations were completed, we parameterized our model using our data set and tested its overall goodness of fit. We used the low *χ* ^2^ value, 0.05 ≤ *p* ≤ 1.00, and 0 ≤ RMSEA ≤ 0.05 (root mean square error of approximation), comparative fit index (CFI > 0.90). Additionally, because some variables were not normal, we confirmed the fit of the model using the Bollen–Stine bootstrap test (the model has a good fit when 0.10 < bootstrap *p* ≤ 1.00), and finally our model attained an acceptable fit by all criteria. With a good model fit, we were free to interpret the path coefficients of the model and their associated *p* values. The SEM analysis was conducted using AMOS 21.0 (SPSS Inc., https://spssau.com/).

## AUTHOR CONTRIBUTIONS

Yang Yang and Yanxing Dou conceived and supervised the study. Yang Yang and Zhijing Xue established the experimental sites. Yang Yang and Baorong Wang collected the samples and analyzed the data. Yunqiang Wang, Shaoshan An, and Scott X. Chang assisted with data analysis. Yang Yang wrote the manuscript with input from all authors.

## CONFLICT OF INTEREST

The authors declare no conflict of interest.

## Supporting information

Supporting information.

Supporting information.

## Data Availability

All the data needed to evaluate the conclusions in the paper are presented in the paper and/or the supplementary materials. The 16S and ITS sequences were submitted to the SRA of the NCBI under accession numbers PRJNA600081 and PRJNA600087. The data and scripts used are saved in GitHub https://github.com/yangyangnature/Soil-microbial-life-history-strategies-in-restored-grasslands. Supplementary materials (figures, tables, scripts, graphical abstract, slides, videos, Chinese translated version and updated materials) may be found in the online DOI or iMeta Science http://www.imeta.science/.
