# Supplementary material for: Deciphering factors driving soil microbial life‐history strategies in restored grasslands
Source: Imeta. 2022 Dec 4;2(1):e66. doi: 10.1002/imt2.66 (PMC10989924; doi:10.1002/imt2.66)
Supplement: Supplementary file 1 — Supporting information. [file IMT2-2-e66-s002.doc]

**Supporting Information for**

**Deciphering** **factors driving soil microbial** **life-history strategies in** **restored grasslands**

^1^ *State Key Laboratory of Loess and Quaternary Geology,* *Institute of Earth Environment,* *Chinese Academy of Sciences, Xi'an, 710061, China*

^2^ *Chinese Academy of Sciences Center for Excellence in Quaternary Science and Global Change, Xi'an, 710061, China*

^3^ *National Observation and Research Station of Earth Critical Zone on the Loess Plateau in* *Shaanxi, Xi’an, 710061, China*

^4^ *State Key Laboratory of Soil Erosion and Dryland Farming on the Loess Plateau, Northwest A&F University, Yangling 712100, China*

^5^ *College of Geography and Tourism, Shaanxi Normal University, Xi 'an 710119, China*

^6^ *Department of Renewable Resources, University of Alberta, Edmonton T6G 2E3, Canada*

** Corresponding author. Yunqiang Wang, State Key Laboratory of Loess and Quaternary Geology, Institute of Earth Environment, Chinese Academy of Sciences, Xi'an, 710061, China. *Email address:* [wangyq@i](mailto:shan@ms.iswc.ac.cn)eecas.cn

** Corresponding author. Shaoshan An, State Key Laboratory of Soil Erosion and Dryland Farming on the Loess Plateau, Northwest A&F University, Shaanxi, 712100, China. *Email address:* [shan@ms.iswc.ac.cn](mailto:shan@ms.iswc.ac.cn)

**This file includes: Figure S1 to S4**


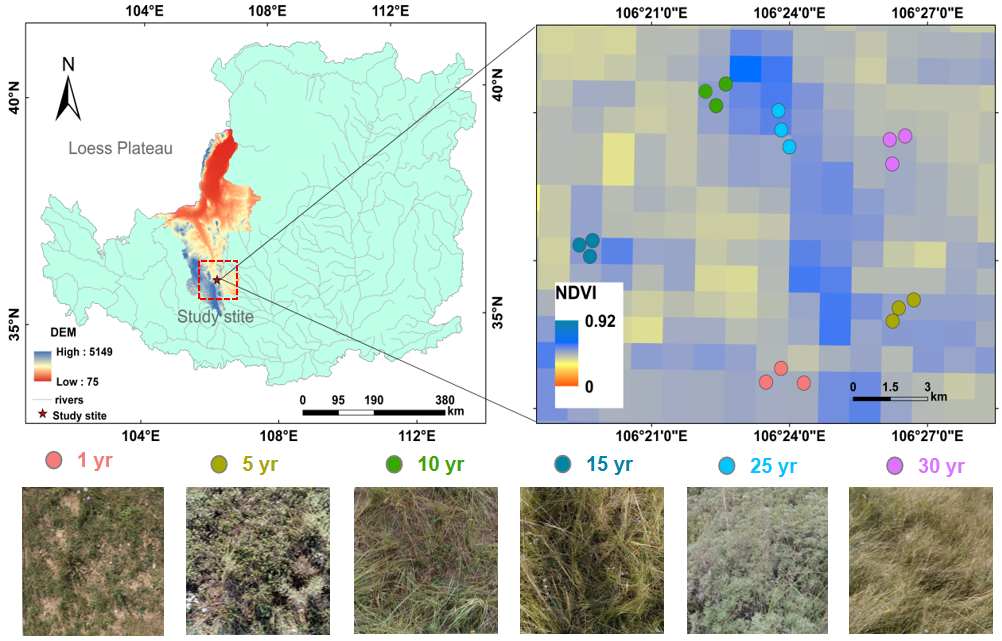


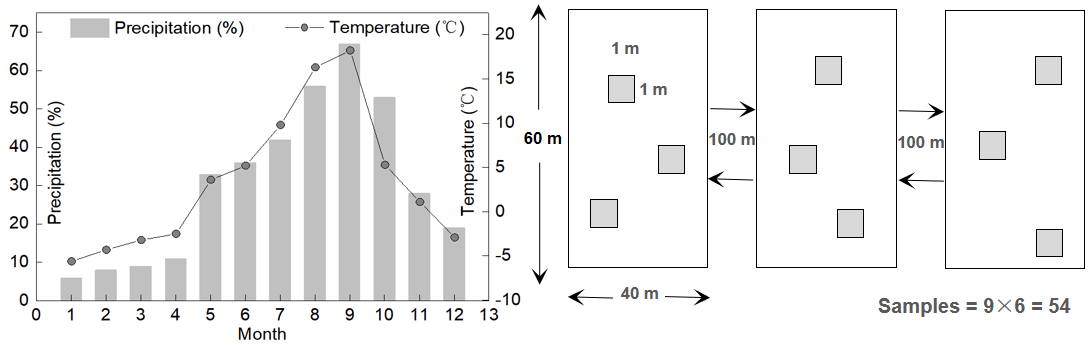


**Figure S1.** Map and experimental design of the study area in restored grasslands on the Loess Plateau, China (The map was created by ArcGIS 9.3 software.


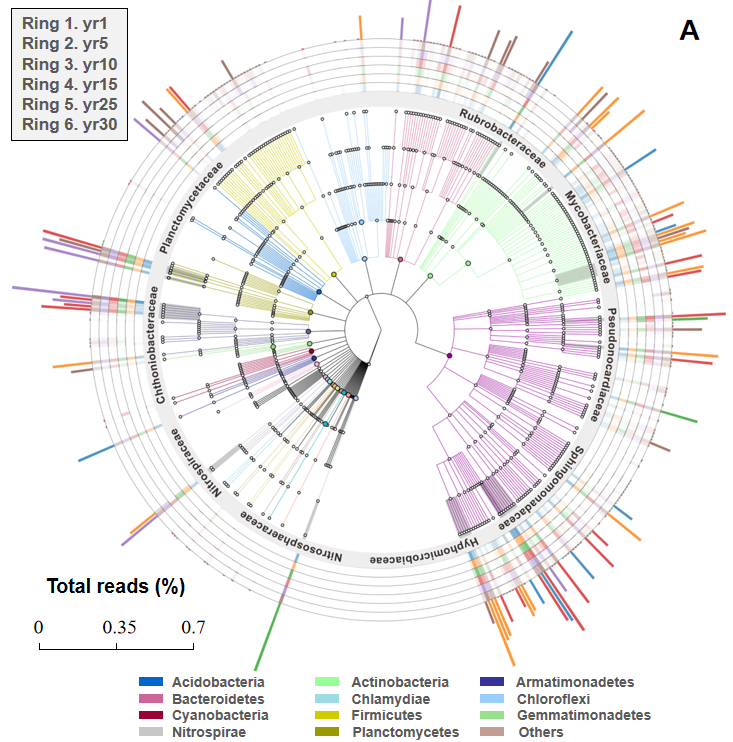

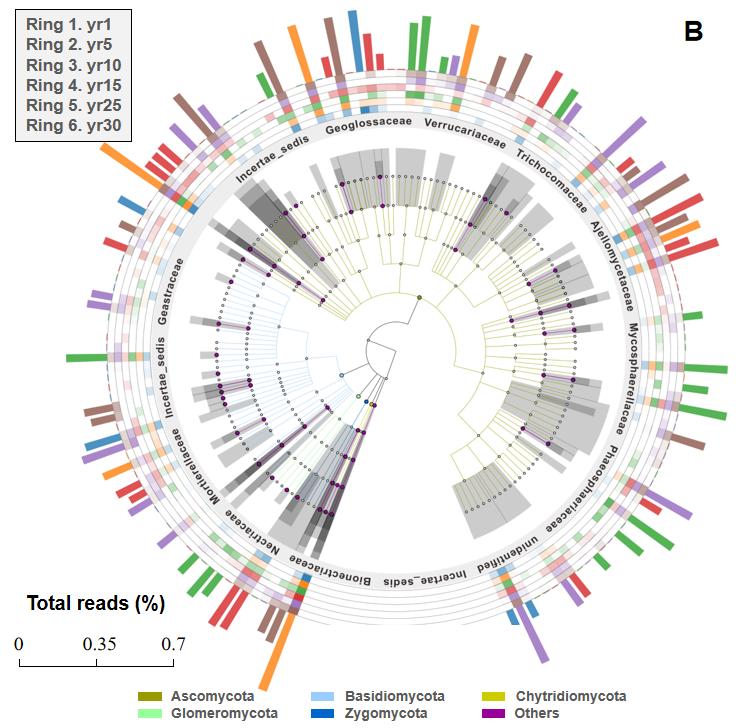


**(A)**

**(B)**

**Figure S2.** Phylogenetic tree including the taxonomic information on dominant soil bacterial (A) and fungal (B) phylotypes. Phylogenetic distribution of the dominant phylotypes. The rings from innermost to outermost indicate 1-, 5-, 10-, 15-, 25- and 30 restoration year, respectively. The bars on the outermost rings indicates, for each phylotype, whether there is a representative isolate and a genome match at the ≥97% 16S and ITS rRNA gene sequence similarity level. The coloring on the outermost ring highlights the distribution of environmental preferences for all phylotypes.


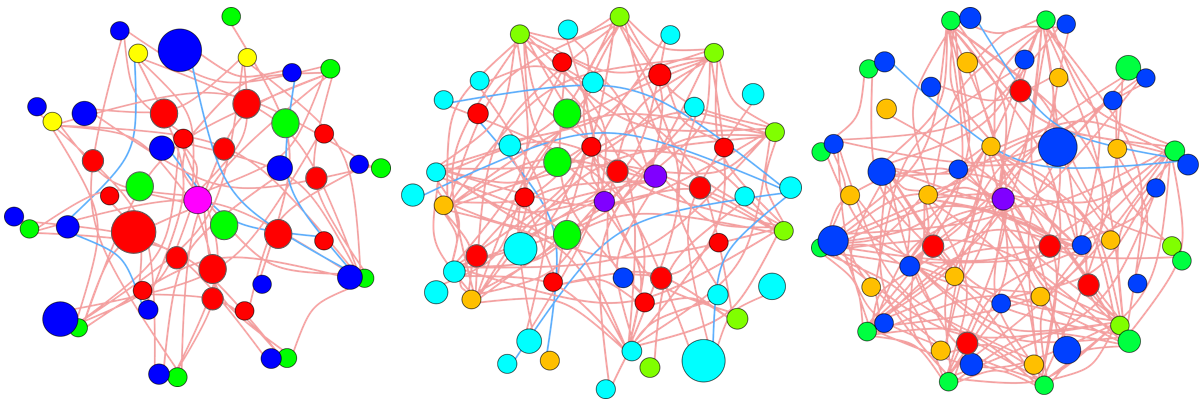

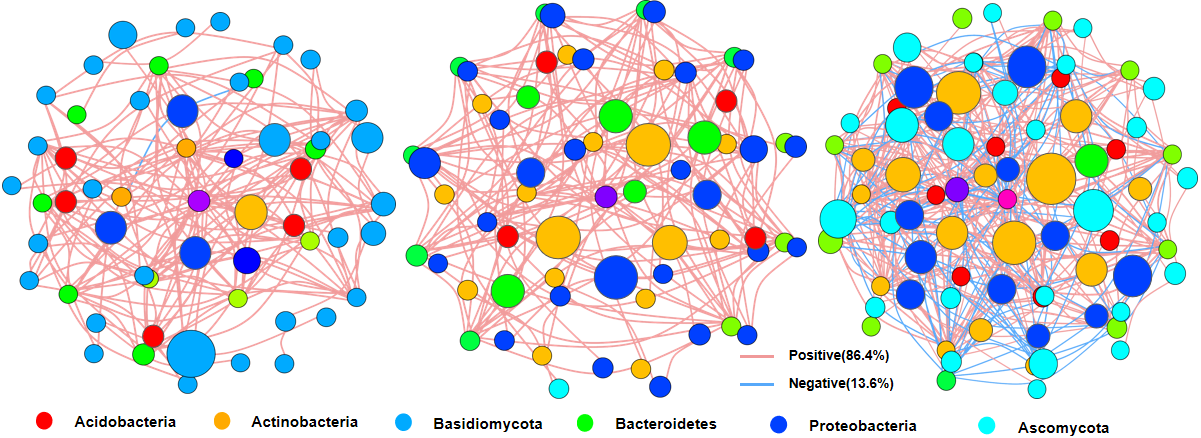


**yr1**

**yr25**

**yr15**

**yr30**

**yr10**

**yr5**

**Figure S3.** Co-occurrence networks and robustness analysis for microbial communities during grassland restoration. In microbial networks, nodes represent individual OTUs whose color and size are positively correlated with the node degree. Network nodes are colored by their affiliated modules. Each edge connecting two nodes represents a significant Spearman correlation (*r* > 0.75 and *P* < 0.05), which red lines indicate as positive correlations, and blue lines indicate as negative correlations.


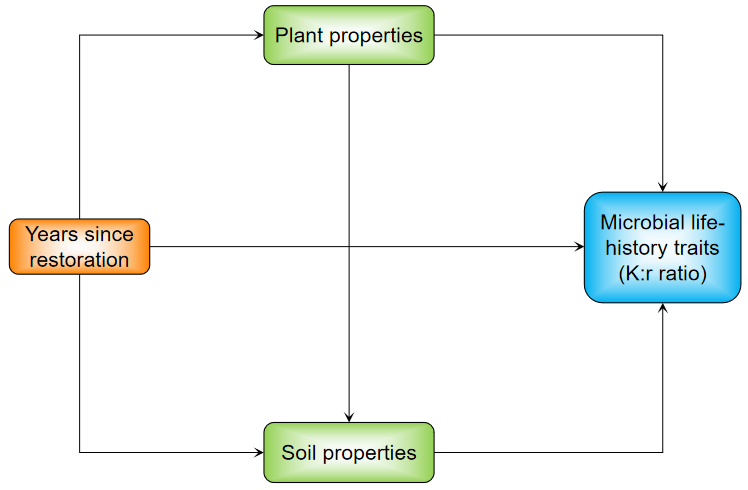


**Figure S4.** The priori structural equation model of plant and soil properties on microbial life-history traits.
